# ProGenFixer: An Ultrafast and Accurate Tool for Correcting Prokaryotic Genome Sequences Using a Mapping-Free Algorithm

**DOI:** 10.34133/csbj.0183

**Published:** 2026-08-25

**Authors:** Lifu Song, Mei Wang, Xiaoping Liao, Junli Wu, Zhenkun Shi, Ruoyu Wang, Haoran Li, Lulu Liu, Botao He, Xiaomeng Ni, Qinggang Li, Jinshan Li, Hongwu Ma, Ping Zheng, Jibin Sun, Yanhe Ma

**Affiliations:** ^1^ College of Biotechnology, Tanjin University of Science and Technology, Tianjin 300457, China.; ^2^ Tianjin Institute of Industrial Biotechnology, Chinese Academy of Sciences, Tianjin 300308, China.; ^3^ National Center of Technology Innovation for Synthetic Biology, Tianjin 300308, China.; ^4^ University of Chinese Academy of Sciences, Beijing 100049, China.; ^5^ State Key Laboratory of Engineering Biology for Low-Carbon Manufacturing, Tianjin 300308, China.; ^6^ Department of Strategic and Integrative Research, Tianjin Institute of Industrial Biotechnology, Chinese Academy of Sciences, Tianjin 300308, China.; ^7^ Institute of Microbiology, Chinese Academy of Sciences, Beijing 100101, China.

## Abstract

Although several tools exist for prokaryotic genome correction, most depend on external read mappers that impose computational overhead and usability barriers, leaving a need for fast, accurate, standalone solutions. We present ProGenFixer, a mapping-free tool that identifies and corrects errors in prokaryotic genomes using next-generation sequencing data. ProGenFixer compares *k*-mer profiles between an input genome and sequencing reads to pinpoint discrepancies, then applies a local assembly-based algorithm with a conservative correction strategy suited to refining high-quality reference genomes. In benchmarking, ProGenFixer ran over 5× faster than the existing tools tested while maintaining higher accuracy. Against slower but highly accurate tools, it showed comparable accuracy while running >17× faster, with better performance on long indels (>10 bp). ProGenFixer is designed primarily for conservative updating of already curated reference sequences rather than aggressive polishing of draft assemblies; accordingly, it does not automatically replace a reference base when substantial read support exists for both the reference and alternate alleles. Comparisons using 3 real bacterial resequencing datasets showed that most tool-specific disagreements occurred at mixed-support or low-depth sites, emphasizing that correction aggressiveness and accuracy are not equivalent in this application. ProGenFixer is implemented in C and runs as a standalone program with no external dependencies. The software is freely available at https://github.com/Scilence2022/ProGenFixer. A companion web platform is available at https://progenfixer.biodesign.ac.cn.

## Introduction

The accuracy of genome sequences is becoming increasingly important to a wide array of biological research and applications, ranging from understanding microbial evolution and function to developing novel antimicrobial therapies and biotechnological processes [1–3]. Next-generation sequencing (NGS) has made whole-genome sequencing rapid and inexpensive. However, the massive genome sequences generated by these technologies are inherently prone to errors arising from various sources during library preparation, sequencing, and data processing. Additionally, rapidly growing bacterial cells are prone to replication errors, which can further introduce additional mutations in genome sequences. Since errors within coding regions, such as insertions and deletions (indels) which can shift reading frames, and substitutions that can alter key residues, can significantly impact protein function, correcting these errors in the genome sequence is critical to downstream phenotypic analyses. Therefore, genome sequence correction, also called genome polishing, improves the accuracy of genomic data and the conclusions drawn from it [4–6].

The primary applications for such correction tools are to polish *de novo* genome assemblies and to correct existing genome sequences based on resequencing data. For instance, a draft genome assembled from long reads can be polished using high-accuracy short reads to fix sequencing errors [4–12]. Alternatively, a published genome sequence for a laboratory strain can be corrected to reflect mutations that have accumulated during cultivation, using short-read sequencing data from the current stock. While these 2 applications are technically similar, they operate under slightly different assumptions. Polishing a *de novo* assembly assumes that the initial sequence is error-prone, and corrections are made whenever resequencing data provides stronger support for an alternative. In contrast, when updating a high-quality, published reference genome of a lab strain, the reference is presumed to be accurate. Therefore, corrections should be made more conservatively, only when there is very strong evidence of an accumulated mutation. The distinction underscores that these 2 tasks require different correction strategies, as an aggressive approach suitable for a draft assembly risks corrupting an already high-quality reference.

This conservative prior is especially important when the read evidence is heterogeneous. Simultaneously abundant reference and alternate alleles can indicate within-sample heterogeneity, a composite or chimeric isolate, contamination, or ambiguous mapping in repetitive sequence. In this setting, retaining the curated base and flagging the locus for review is preferable to forcing a consensus change.

Numerous genome sequence correction tools have been developed, employing diverse algorithmic strategies [4–15]. These tools can broadly be categorized into mapping-based and mapping-free methods. Mapping-based methods typically rely on aligning sequencing reads back to a reference genome sequence to identify discrepancies and correct errors. Recent advancements have led to the development of fast read aligners that utilize hash functions, advanced indices, and seeding techniques to accelerate the alignment process. Widely used mapping-based pipelines, such as those combining Burrows–Wheeler Aligner (BWA) [16] or minimap2 [17] with variant callers like Genome Analysis Toolkit (GATK) [18] or BCFtools [19], as well as dedicated polishers including Pilon [7], Polypolish [8], and POLishing by Calling Alternatives (POLCA) [9], have become standard components of genome-finishing workflows. Despite these advantages, mapping-based genome correction can be computationally expensive and time-consuming, as the read-alignment step dominates the overall runtime and typically requires auxiliary sorting and indexing of the resulting alignment files. In contrast, mapping-free methods operate by comparing *k*-mer profiles (short subsequences of length *k*) rather than aligning full-length sequences. The concept of *k*-mers, which has broad applications such as sequence alignment, genome assembly, and DNA data storage [20,21], plays a central role in these methods. Mapping-free algorithms are computationally inexpensive and can run substantially faster than mapping-based methods [20]. Several mapping-free polishing tools have been developed on this principle, including ntEdit [13], which uses a Bloom filter to evaluate *k*-mer support, Merfin [12], which leverages *k*-mer multiplicity to validate candidate variants, and Jasper [4]. Mapping-free methods also have limitations. For instance, accurately correcting errors within repetitive regions of the genome poses a significant challenge for these methods, as the *k*-mers derived from such regions are often ambiguous and occur in high frequencies, and the choice of *k*-mer length and coverage thresholds strongly influences the balance between sensitivity and precision, such that a single fixed threshold may be insufficient to distinguish genuine sequencing errors from low-coverage but correct regions [4,11,14,15].

Prokaryotic genomes are typically simpler than eukaryotic ones (haploid, smaller and fewer repeats), making variation analysis more straightforward [22]. Furthermore, many general-purpose genome correction tools are developed with the complexity of eukaryotic genomes in mind, which can introduce unnecessary computational overhead when applied to the relatively simpler genomes of prokaryotes. Moreover, while these tools can be applied to both draft polishing and reference updating, few are specifically designed with the conservative strategy required for accurately correcting an established, high-quality reference genome. Additionally, these tools often impose further burdens on users by requiring dependencies on external read mapping and sorting software, which exacerbates both computational costs and installation difficulties. Thus, there is a clear need for an efficient and user-friendly tool specifically tailored for the rapid correction of prokaryotic genome sequences.

To address this need, we present ProGenFixer, a mapping-free tool engineered for rapid and accurate prokaryotic genome correction. ProGenFixer rapidly compares *k*-mer profiling of NGS data with the ordered *k*-mer array of an input genome to detect low-quality regions. It then estimates the altered sequence content region by region using a local assembly algorithm. Unlike previous studies that use a single coverage threshold to eliminate low-coverage *k*-mers, ProGenFixer employs differential thresholds when detecting low-quality regions and estimating altered sequence content, balancing sensitivity and precision. ProGenFixer was implemented in multithreaded C for maximum efficiency. Crucially, ProGenFixer operates as a self-contained program that requires no external read-mapping or file-sorting dependencies, which simplifies installation and reduces the computational overhead typically associated with mapping-based workflows. To accommodate the differing requirements of de novo assembly polishing and high-quality reference updating, ProGenFixer additionally supports an adjustable correction stringency, enabling either aggressive correction of error-prone drafts or conservative updating of established reference genomes. In benchmarks against Jasper [4], NextPolish [5], Pypolca [6], and BWA+GATK [16,18], ProGenFixer ran more than 5 times faster while matching or exceeding their accuracy, with a clear advantage on long indels (>10 bp). These properties make it useful both for finishing genome assemblies and for maintaining laboratory reference strains.

## Results and Discussion

### Design and implementation of ProGenFixer

**Fig. 1.**
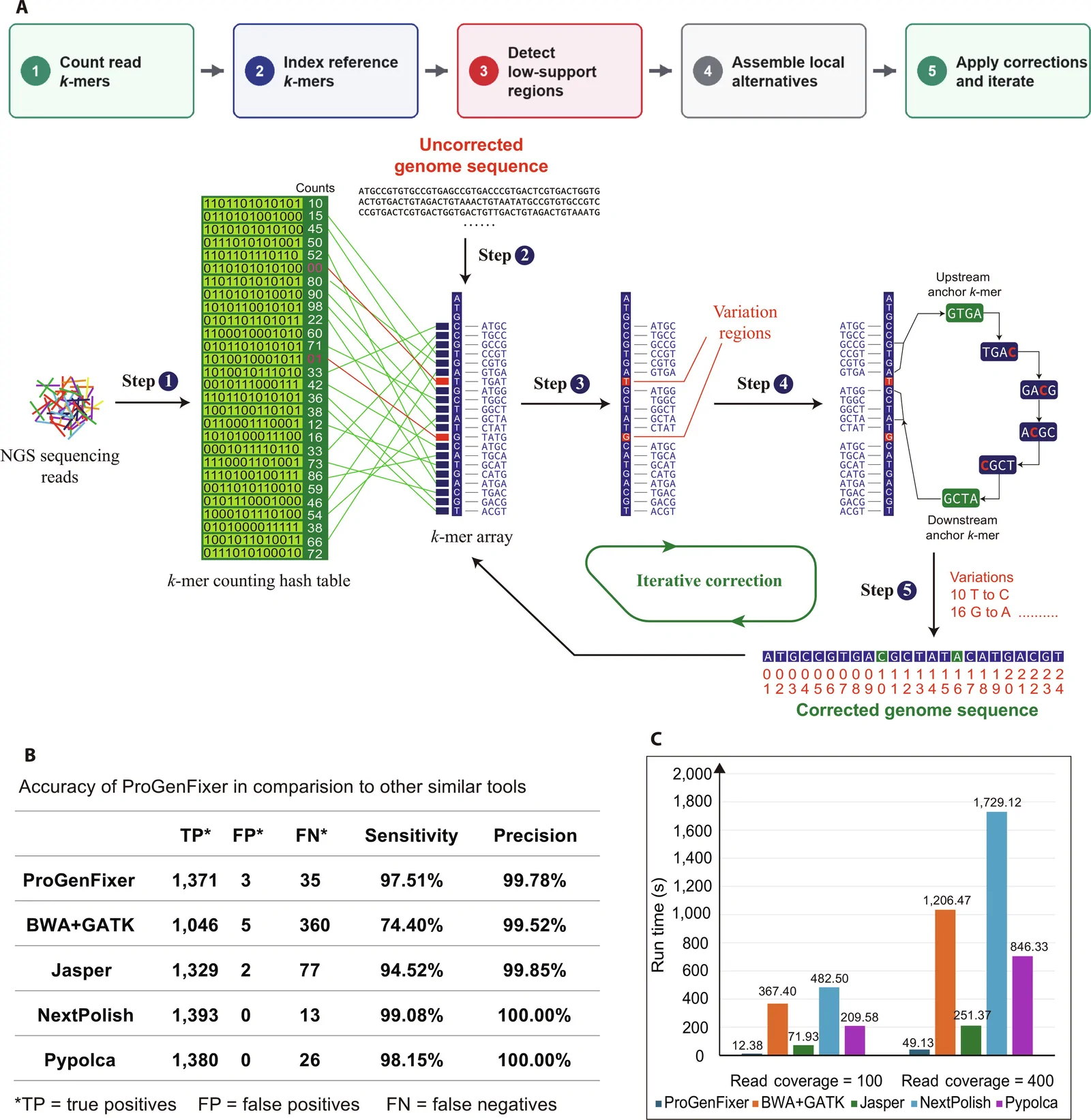
Workflow, accuracy, and speed of ProGenFixer in comparison with existing genome correction tools: (A) Schematic overview of the ProGenFixer workflow. Step 1: Construct a *k*-mer counting hash table from next-generation sequencing (NGS) reads. Step 2: Encode the input genome sequence into an ordered *k*-mer array, mapping each *k*-mer to its genomic position. Step 3: Identify potential variation regions (highlighted in red) by checking the support for input genome *k*-mers within the read-derived *k*-mer hash table. Step 4: Determine the corrected sequence content within variation regions using a local assembly-based approach. Step 5: Produce the final corrected genome sequence incorporating identified variations (illustrative example shows 10T→C and 16G→A changes). This corrected sequence can serve as the new input for subsequent correction rounds (iterative correction). (B) Accuracy metrics (TP = true positives, FP = false positives, FN = false negatives, sensitivity, precision) of ProGenFixer compared to other tools for correcting a mixture of substitutions and indels. (C) Speed comparison of ProGenFixer and other genome correction tools. The bar graph displays the run time (seconds) required to correct the *E. coli* MG1655 genome using various tools (ProGenFixer, Burrows–Wheeler Aligner [BWA] + Genome Analysis Toolkit [GATK], Jasper, NextPolish, and Pypolca) with simulated read coverages of 100× and 400×. Runtimes represent the average of 3 independent replicates; SDs are provided in Table S2 (too small to visualize as error bars).

As illustrated in Fig. 1A, ProGenFixer operates using an iterative workflow consisting of 5 major steps, 4 of which are repeated in each iteration. Fundamentally, ProGenFixer refines an input genome sequence by analyzing *k*-mer frequencies from the NGS reads and comparing them against *k*-mers derived from the input genome sequence. This comparison pinpoints regions with discrepancies or low *k*-mer support, marking them as potential variation regions. To resolve these flagged areas, the tool constructs alternative sequences by navigating a *k*-mer graph weighted by *k*-mer counts, ultimately selecting the most probable correction. The resulting corrected genome sequence then serves as the input sequence for the next iteration of correction, allowing for progressively enhanced accuracy. The 5 steps are detailed as follows:

The conservative decision rule is central to ProGenFixer’s intended use. The input reference is treated as a curated prior rather than as an error-rich draft. Candidate regions can be detected sensitively, but a sequence change requires stronger and internally consistent *k*-mer support. When the reference and an alternative are both substantially represented, ProGenFixer favors retaining the input sequence instead of converting a heterogeneous signal into a single consensus allele. Such a noncall is an intentional safeguard for reference maintenance, although the site can still be reviewed or validated with independent evidence.

Step 1. ProGenFixer counts the *k*-mers of NGS reads using a simple and efficient hash table. This choice ensures that large datasets are processed swiftly while maintaining the accuracy of *k*-mer counts. Given the relatively small size of bacterial genomes, the advantages of the Bloom filter structure are limited, although it has been shown to be an effective strategy for reducing memory usage. As a result, the Bloom filter structure is not implemented in ProGenFixer. Additionally, the *k*-mer counting process is implemented using a multithreaded architecture to enhance performance.

Step 2. The input genome sequence is encoded into ordered arrays of *k*-mers, with each index correlating to the *k*-mer’s position within the genome sequence. This representation enables ProGenFixer to track the precise position of each *k*-mer in the input genome, which is crucial for accurately detecting low-quality regions with potential variations in the next step.

Step 3. ProGenFixer checks each *k*-mer in the *k*-mer array derived from the input genome sequence to assess its presence in the *k*-mer counter table generated from the NGS reads. *K*-mers that are either absent or have coverage values below a specified threshold in the counter table are flagged as potential variation regions, indicative of errors. To minimize amplification and sequencing errors, previous studies often implement a uniform *k*-mer coverage threshold to filter out low-coverage *k*-mers. In contrast, ProGenFixer utilizes differential thresholds during 2 consecutive processes: identifying variation regions and estimating the altered sequence contents (i.e., variation calling). A lower coverage threshold is used to identify candidate variation regions, maximizing sensitivity, while a higher threshold is applied when estimating the corrected sequence content, maximizing precision. This dual-threshold strategy raises the likelihood that flagged regions are genuine error sites, the risk of incorrect corrections. It is especially useful when updating high-quality reference genomes, where a conservative approach avoids introducing errors into an already accurate sequence.

Step 4. For each variation region identified with potential errors, ProGenFixer estimates alternative sequences by traversing the *k*-mer graph. The algorithm selects the most likely path based on the *k*-mer counter table. Specifically, ProGenFixer first merges variation regions that are within a distance not greater than the *k*-mer size. Next, the algorithm extends the region by including upstream and downstream high-coverage anchor *k*-mers (those present in the input genome’s *k*-mer array and adjacent to the variation region), as illustrated in Fig. 1A. Two criteria must be satisfied for a *k-*mer to be selected as an anchor *k*-mer: First, it must not be present multiple times in the input genome sequence; second, the corresponding coverage in the *k*-mer counter table must be higher than a specified threshold for estimating the altered sequence contents. If the nearby *k*-mers do not meet the anchor criteria, ProGenFixer will continue extending the region outward from the variation site, searching for the nearest *k*-mer that satisfies the criteria. Finally, the alternative sequence is estimated by traversing the *k*-mer graph using the counter table.

Step 5. ProGenFixer corrects the input genome sequence based on the variations estimated in the previous step. The corrected genome sequence then becomes the new input for the next iteration of corrections, as needed.

### Accuracy and speed of ProGenFixer in comparison with existing tools

We evaluated ProGenFixer’s performance using simulated data based on the genomes of 2 industrially important strains: *Escherichia coli* MG1655 (LR881938.1) and *Corynebacterium glutamicum* ATCC 13032 (BA000036.3) [1,2]. Results were highly consistent between the 2 genomes; therefore, we present representative results for *E. coli* MG1655 here, with complete simulation results available in Fig. S1 and Tables S1 to S4.

First, we assessed the performance of ProGenFixer in handling a mixture of substitutions and insertions/deletions (indels). Using dwgsim v0.1.15 [23], we simulated 150-bp paired-end NGS reads at 100× and 400× coverage. The simulation incorporated a mutation rate (*-r*) of 0.3‰ (parts per thousand), with 10% of mutations being indels and an indel extension probability of 0.3. To benchmark ProGenFixer, we compared its performance against established tools: BWA+GATK, Jasper, NextPolish, and Pypolca. As shown in Fig. 1B, ProGenFixer achieved high sensitivity (97.51%) and precision (99.78%), comparable to the top-performing tools. ProGenFixer was also considerably faster, correcting the *E. coli* genome with 100× reads in approximately 12.93 s, whereas the fastest competitor, Jasper, required 71.93 s. This represents a greater than 5-fold speed increase over the other tested tools (Fig. 1C). Similar performance gains were observed in simulations using high-coverage (400×) reads (Fig. 1C).

Next, we evaluated ProGenFixer’s performance specifically on substitutions and indels independently. To assess ProGenFixer’s capability of substitution correction, we simulated 100× coverage NGS reads with varying substitution rates (0.01%, 0.03%, 0.05%, 0.07%, 0.09%, and 0.1%). These reads were then processed using ProGenFixer and the comparison tools. As detailed in Table 1, ProGenFixer maintained high sensitivity (97.54% to 98.49%) and precision (99.56% to 100.00%) across all substitution rates, comparable to NextPolish and Pypolca, the best performers in this test.

**Table 1.** Performance of ProGenFixer in handling substitutions. NGS reads with a coverage of 100× were simulated using dwgsim (version 0.1.15) with the *E. coli* MG1655 genome sequence (LR881938.1). The expected mutation rates were adjusted using the *-r* option.

Metric	ProGenFixer	BWA+GATK	Jasper	NextPolish	Pypolca	Mutation rate
TP	456	370	453	460	456	0.01%
FP	0	3	0	0	0	
FN	7	93	10	3	7	
Sensitivity	98.49%	79.91%	97.84%	99.35%	98.49%	
Precision	100%	99.20%	100%	100%	100%	
TP	1,332	1,091	1,331	1,352	1,340	0.03%
FP	4	0	1	0	0	
FN	31	272	32	11	23	
Sensitivity	97.73%	80.04%	97.65%	99.19%	98.31%	
Precision	99.70%	100%	99.92%	100%	100%	
TP	2,319	1,921	2,311	2,352	2,333	0.05%
FP	2	3	2	0	0	
FN	43	441	51	10	29	
Sensitivity	98.18%	81.33%	97.84%	99.58%	98.77%	
Precision	99.91%	99.84%	99.91%	100%	100%	
TP	3,195	2,607	3,188	3,238	3,210	0.07%
FP	13	2	3	0	0	
FN	64	652	71	21	49	
Sensitivity	98.04%	79.99%	97.82%	99.36%	98.50%	
Precision	99.59%	99.92%	99.91%	100%	100%	
TP	4,090	3,337	4,082	4,174	4,127	0.09%
FP	18	8	5	0	0	
FN	103	856	111	19	66	
Sensitivity	97.54%	79.59%	97.35%	99.55%	98.43%	
Precision	99.56%	99.76%	99.88%	100%	100%	
TP	4,448	3,604	4,442	4,521	4,480	0.1%
FP	9	7	2	4	0	
FN	108	952	114	35	76	
Sensitivity	97.63%	79.10%	97.50%	99.23%	98.33%	
Precision	99.80%	99.81%	99.95%	99.91%	100%	

TP, true positives; FP, false positives; FN, false negatives; NGS, next-generation sequencing; BWA, Burrows–Wheeler Aligner; GATK, Genome Analysis Toolkit

We also systematically evaluated ProGenFixer’s performance in correcting indels of varying expected lengths (ranging from ~1.1 to ~20 bp), comparing it against other tools (Table 2). ProGenFixer maintained high sensitivity (96.52% to 99.34%) and precision (99.33% to 100.00%) across all tested indel lengths. In contrast, the performance of some other tools degraded significantly as indel length increased. Jasper’s sensitivity fell sharply for longer indels, from 86.94% to 6.72%. While NextPolish and Pypolca performed well on shorter indels, their sensitivity also decreased for the longest indels tested (~10 and ~20 bp). BWA+GATK consistently exhibited low sensitivity across all lengths. For the longest simulated indels (~20 bp), ProGenFixer still retained high sensitivity (96.52%) and precision (99.33%), unlike the other tools tested.

**Table 2.** Performance of ProGenFixer in handling indels of varying lengths. NGS reads were simulated using dwgsim (version 0.1.15) at a coverage of 100× using the *E. coli* MG1655 genome sequence (LR881938.1). The expected indel lengths were varied using the -X option, which controls the probability of indel extension. The calculation method for determining the expected indel lengths associated with different -X values is detailed in Materials and Methods.

Metric	ProGenFixer	BWA+GATK	Jasper	NextPolish	Pypolca	Indel length
TP	1,210	225	1,078	1,232	1,221	~1.11 bp *-X 0.1*
FP	2	2	1	0	0	
FN	30	1,015	162	8	19	
Sensitivity	97.58%	18.15%	86.94%	99.35%	98.47%	
Precision	99.83%	99.12%	99.91%	100.00%	100.00%	
TP	1,290	291	909	1,314	1,301	~1.43 bp *-X 0.3*
FP	3	5	2	2	0	
FN	35	1,034	416	11	24	
Sensitivity	97.36%	21.96%	68.60%	99.17%	98.19%	
Precision	99.77%	98.31%	99.78%	99.85%	100.00%	
TP	1,325	285	679	1,354	1,339	~2 bp *-X 0.5*
FP	2	1	2	0	0	
FN	38	1,078	684	9	24	
Sensitivity	97.21%	20.91%	49.82%	99.34%	98.24%	
Precision	99.85%	99.65%	99.71%	100.00%	100.00%	
TP	1,286	287	405	1,306	1,298	~3.33 bp *-X 0.7*
FP	1	5	6	0	0	
FN	28	1,027	909	8	16	
Sensitivity	97.87%	21.84%	30.82%	99.39%	98.78%	
Precision	99.92%	98.29%	98.54%	100.00%	100.00%	
TP	1,281	275	140	1,304	1,224	~10 bp *-X 0.9*
FP	5	5	4	0	34	
FN	38	1,044	1,179	15	95	
Sensitivity	97.12%	20.85%	10.61%	98.86%	92.80%	
Precision	99.61%	98.21%	97.22%	100.00%	97.30%	
TP	1,193	259	83	1,135	1,050	~20 bp *-X 0.95*
FP	8	6	1	32	30	
FN	43	977	1,153	101	186	
Sensitivity	96.52%	20.95%	6.72%	91.83%	84.95%	
Precision	99.33%	97.74%	98.81%	97.26%	97.22%	

TP, true positives; FP, false positives; FN, false negatives; NGS, next-generation sequencing; BWA, Burrows–Wheeler Aligner; GATK, Genome Analysis Toolkit

### Validation on real bacterial resequencing datasets

**Fig. 2.**
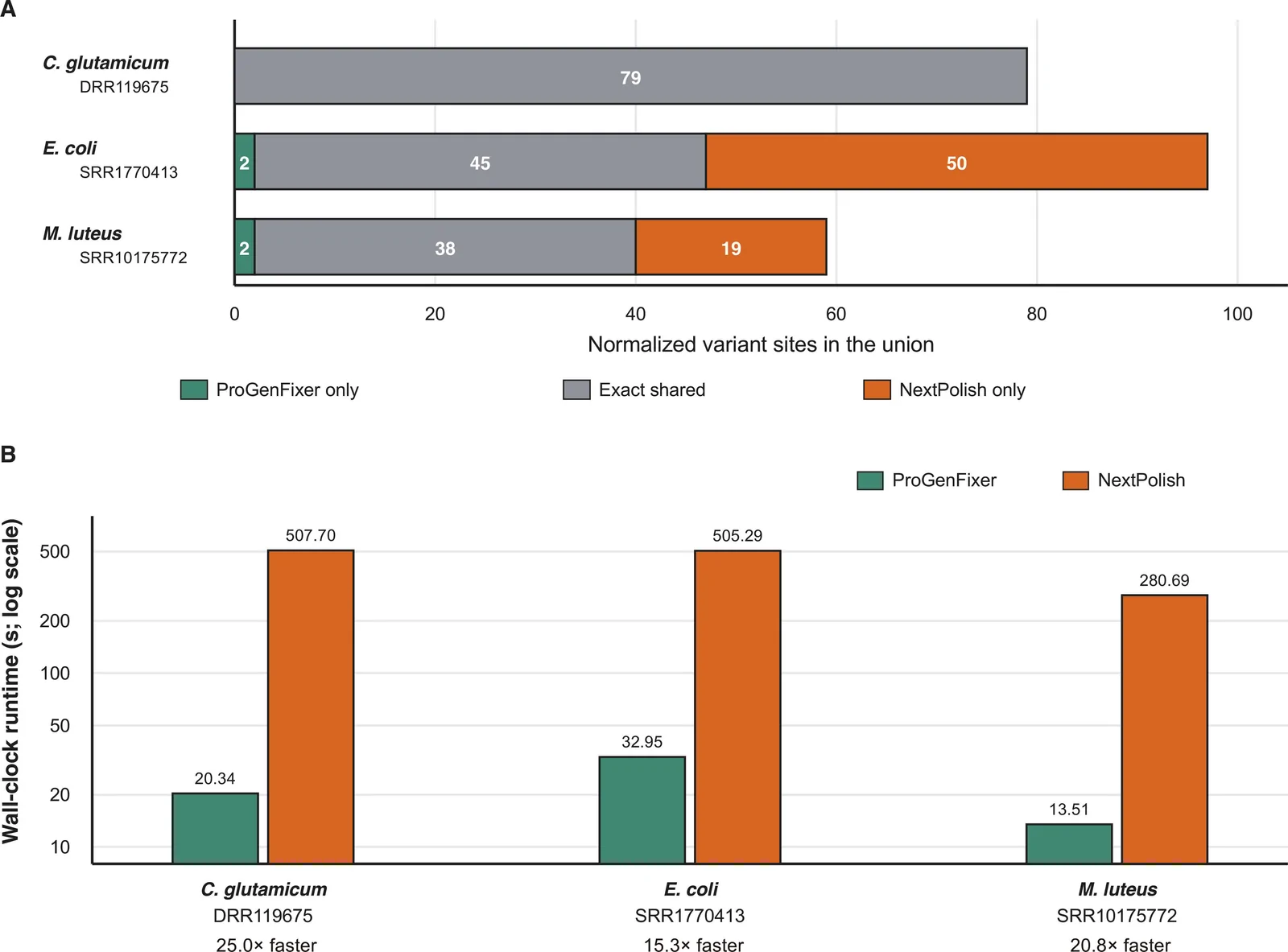
Real-data comparison of ProGenFixer and NextPolish. (A) Exact overlap of normalized variant calls after independently aligning each corrected sequence to its original reference. Bars show variants shared by ProGenFixer and NextPolish and variants specific to either tool. After normalization, all 79 DRR119675 variants were shared; SRR1770413 had 45 shared, 2 ProGenFixer-specific, and 50 NextPolish-specific variants; and SRR10175772 had 38 shared, 2 ProGenFixer-specific, and 19 NextPolish-specific variants. The large NextPolish-specific counts (50 and 19) predominantly reflect sites with coexisting reference and alternate alleles or low-filtered read depth rather than homogeneously supported corrections (see main text and Table 3), consistent with ProGenFixer’s conservative reference-maintenance objective. (B) Wall-clock runtime using identical paired-end reads and matched references; the y-axis is logarithmic.

To evaluate the intended reference-update application beyond simulated benchmarks, we applied ProGenFixer and NextPolish to 3 independent paired-end Illumina resequencing datasets: *Corynebacterium glutamicum* ATCC 13032 (DRR119675; reference BA000036.3), *Escherichia coli* K-12 MG1655 (SRR1770413; U00096.3), and the high-GC bacterium *Micrococcus luteus* NCTC 2665 (SRR10175772; NZ_LS483396.1). The corrected sequences from each tool were aligned independently to the corresponding original reference, and exact normalized variant overlaps and site-level read support were compared (Fig. 2 and Table 3). Because no independent truth assembly was available, tool-specific changes were interpreted as different correction decisions rather than as false positives or false negatives.

**Table 3.** Comparison of ProGenFixer and NextPolish using 3 real bacterial resequencing datasets. Final corrected sequences were independently aligned to the corresponding original reference. Variant counts are normalized differences relative to that reference, not accuracy estimates against an independent truth assembly. In the intended reference-update setting, retaining the curated reference allele at a mixed-support site is a conservative outcome rather than an automatic false negative. Note: The 2 *E. coli* deletion intervals had mean depths of 0.40× and 0.99×, compared with approximately 26 to 57× in their flanks. When both the reference and alternate alleles have substantial read support, the evidence may reflect sample heterogeneity, a composite or chimeric isolate, contamination, or ambiguous mapping; such sites should be retained or flagged for review rather than automatically correct.

Dataset	Reference (size; GC)	Runtime (s) ProGenFixer / NextPolish	Variants ProGenFixer / NextPolish	Exact shared	Unique ProGenFixer / NextPolish
*C. glutamicum* DRR119675	BA000036.3 3.31 Mb; 53.81%	20.34 / 507.70	79 / 79	79	0 / 0
*E. coli* SRR1770413	U00096.3 4.64 Mb; 50.79%	32.95 / 505.29	47 / 95	45	2 / 50
*M. luteus* SRR10175772	NZ_LS483396.1 2.50 Mb; 73.00%	13.51 / 280.69	40 / 57	38	2 / 19

**Fig. 3.**
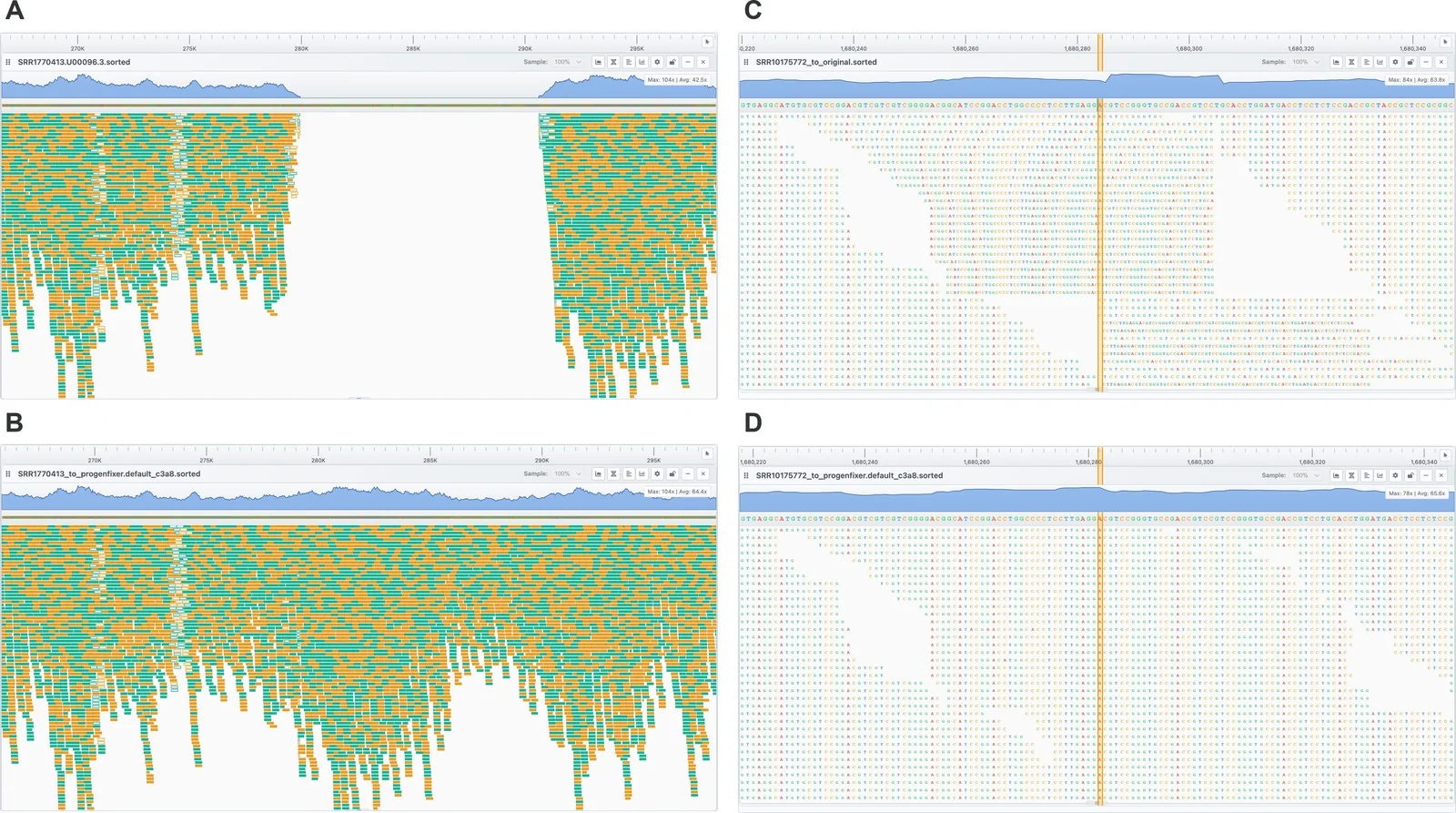
Representative read-mapping support for ProGenFixer-corrected deletion and insertion events in real Illumina datasets. Illumina reads were mapped to the genome sequence before and after ProGenFixer correction and visualized using CodeXomics (https://github.com/Scilence2022/CodeXomics). (A and B) In SRR1770413, the original *E. coli* reference contains a large low/no-coverage interval at U00096.3:279163-290633 with covered flanking regions. After ProGenFixer correction, reads support the new junction across the deleted segment, indicating that this reference interval is absent from the sequenced isolate. (C and D) In SRR10175772, the original *M. luteus* reference shows local mapping discordance consistent with sequence missing at NZ_LS483396.1:1680285. After insertion of ACGTCCGGGTGCCGACCGTC by ProGenFixer, reads align coherently across the corrected locus, supporting the inserted sequence. These examples are representative cases; additional ProGenFixer-specific corrections and read-support details are presented in Figs. S2 to S5 and Table S7.

For *C. glutamicum*, the tools produced final sequences of identical length and shared all 79 variants exactly. The *E. coli* dataset behaved differently. ProGenFixer and NextPolish shared 45 exact variants, but ProGenFixer additionally removed 776- and 11,471-bp reference intervals. Mean read depths within these intervals were 0.40× and 0.99×, respectively, whereas their flanks had approximately 26× to 57× coverage, strongly supporting absence of the segments from the sequenced isolate. Read mapping across the corrected junction confirmed this deletion, with reads spanning the new boundary after ProGenFixer correction (Fig. 3A and B). NextPolish retained these regions and instead reported 50 unique single-nucleotide polymorphisms (SNPs). Of 47 sites with evaluable filtered pileups, only 17 had an alternate-allele majority, and the average counts were approximately 59 alternate and 42 reference reads at approximately 102× depth. This recurrent coexistence of both alleles is not the homogeneous evidence expected for a routine reference-base replacement.

The *M. luteus* dataset provided a high-GC test (73.00% GC) with 99.37% reference coverage at a mean depth of 43.17×. The tools shared 38 exact variants; ProGenFixer and NextPolish had 2 and 19 tool-specific variants, respectively. The ProGenFixer-only SNPs were contradicted by 35 to 39 filtered reads supporting the reference allele. Most NextPolish-only SNPs occurred at low-filtered depth, although NZ_LS483396.1:1,485,256 C>T had 12/12 alternate-supporting reads and position 2,233,494 G>A had 36/63 alternate-supporting reads. The former is a strongly supported alternative, whereas the latter retains substantial support for both alleles. Most remaining disagreements were too low-depth to justify confident replacement of a curated reference base. A representative ProGenFixer insertion in this dataset (ACGTCCGGGTGCCGACCGTC at NZ_LS483396.1:1,680,285) was likewise supported by coherent read alignment across the corrected locus (Fig. 3C and D). These results demonstrate that tool-specific corrections in real datasets require site-level read-support assessment.

Across the 3 datasets, most disagreements involved substantial support for both alleles or insufficient read depth. Such sites may arise from a heterogeneous culture, a composite or chimeric strain, contamination, or ambiguous mapping, although these alternatives cannot be distinguished from the present data alone. Under ProGenFixer’s reference-maintenance objective, declining to overwrite these loci is a conservative design outcome and should not be scored as inaccuracy merely because a conventional polisher emitted a change. Conversely, the near-homogeneous alternate support at BA000036.3:3,035,474 and NZ_LS483396.1:1,485,256 identifies cases in which an alternative deserves additional validation.

Across the 3 runs, ProGenFixer completed in 13.51 to 32.95 s, compared with 280.69 to 507.70 s for NextPolish, corresponding to a 15.3- to 25.0-fold runtime advantage (Fig. 2B). The real-data analyses confirm ProGenFixer’s speed and its ability to identify long unsupported reference segments. They also show why reference updating should be judged by whether a change is warranted under the observed evidence, rather than by treating the more aggressive tool’s output as ground truth.

### Establishment of a web-based platform for ProGenFixer

We also developed a web platform that lets users correct a genome sequence by uploading NGS data and an input genome sequence. The platform automatically processes the provided input genome and NGS files and corrects the genome sequence utilizing ProGenFixer. Upon completion of the correction, the server returns the corrected genome sequence in FASTA format, along with a detailed report of the corrected variations in Variant Call Format (VCF). This user-friendly web server can be accessed at: https://progenfixer.biodesign.ac.cn/.

## Conclusion

ProGenFixer is a fast, accurate tool for prokaryotic genome sequence correction. Our benchmarking demonstrates that it operates over 5 times faster than leading tools such as Jasper, NextPolish, and Pypolca, while maintaining comparable sensitivity and precision for error detection and correction; on 3 real bacterial resequencing datasets, it achieved a 15.3- to 25.0-fold runtime advantage over NextPolish. It is particularly accurate on long indels (>10 bp) and reliably detects long, read-unsupported reference regions. These properties make it well suited to prokaryotic genomics.

ProGenFixer is designed for key tasks in prokaryotic genomics, such as polishing draft genome assemblies and, particularly, updating existing high-quality genome sequences with new resequencing data. Its principal application is therefore conservative maintenance of already polished reference sequences rather than maximal alteration of an error-rich draft assembly, a principle that guided the tool’s design to ensure that changes are made only with high confidence. Consistent with this design, most small-variant disagreements in the real-data evaluation were associated with mixed or low-depth evidence; in this setting, retaining the curated reference is an intended safeguard rather than evidence of lower accuracy, and ambiguous sites should be reported for manual or orthogonal validation instead of being forced into a single consensus sequence.

While ProGenFixer exhibits high overall accuracy, its performance is currently less optimal in repetitive genomic regions, leading to a noticeable number of false positives and false negatives. Future development could mitigate this limitation by integrating a local alignment-based strategy specifically designed to resolve these complex sequences, thereby improving accuracy without sacrificing the tool’s core computational efficiency. Because ProGenFixer relies on *k*-mer evidence, extreme base composition may also affect performance: In strongly AT- or GC-skewed genomes, informative *k*-mers become sparser and low-complexity or homopolymeric tracts more frequent, which can reduce the number of confidently correctable sites and increase context ambiguity at those loci. We note that ProGenFixer nonetheless retained high accuracy on the high-GC (73.00%) *M. luteus* dataset, but a systematic evaluation across a wider compositional range remains valuable future work.

The high efficiency of ProGenFixer is particularly relevant in the context of modern synthetic biology, where biofoundry platforms, big data analytics, and artificial intelligence are driving rapid advancements in microbial strain optimization [22,24,25]. This evolving paradigm generates vast numbers of mutant strains, demanding substantial computational resources for genomic analysis. ProGenFixer’s efficient framework directly addresses this challenge, enabling the rapid and precise analysis of large-scale genomic datasets. This throughput can help accelerate the design-build-test-learn cycles of biological engineering in data-driven synthetic biology and biofoundry work.

## Materials and Methods

### Simulation of variations, NGS reads, and calculation of expected length of indels

All variations and associated NGS reads were simulated with dwgsim (v0.1.15) with a range of parameters, allowing for the simulation of different rates of substitutions and indels. The simulation of indels was performed using the -X option, which specifies the probability (*P*) that an indel is extended at each step. When an indel occurs, dwgsim uses a certain probability *P* to determine whether the indel will continue to extend during simulation. The probability of the indel stopping at any moment is 1 − *P*. After each extension, there is still a chance for further extension, making the problem recursive in nature. The average length of an indel can be calculated using a recursive model based on the probability of extension. The length of the indel can be described as a geometric random variable because we are modeling the number of successes (extensions) before the first failure (when the indel stops extending). Let *E*_L_ represent the expected length of an indel. The recursive relationship for the expected length *E*_L_ is as follows:

$$E_{L}=1\times \left(1-P\right)+\left(1+E_{L}\right)\times P$$ (1)

where 1 × (1 − *p*) represents the case where the indel stops after extending once; (1 + *E*_L_) × *P* represents the case where the indel continues to extend by 1 unit and the length is then increased by 1 plus the expected length of any subsequent extensions. By simplifying Eq. (1), we obtain:

$$E_{L}=1/\left(1-P\right)$$ (2)

This formula calculates the average indel length, where *P* is the probability of extending the indel by 1 unit.

### Evaluation framework of simulated data

To assess the accuracy of genome correction tools, we developed an evaluation pipeline using Minimap2, SAMTools, and BCFtools. The pipeline consists of a bash script (compare_genomes.sh) and a python script (vcf_comparison.py). The 2 scripts are provided alongside the source code of ProGenFixer. The bash script implements a comprehensive workflow for identifying genomic variations between 2 genome sequences. The process begins with alignment of the query genome to the input genome sequence using Minimap2 [17]. The resulting alignments are converted to sorted BAM format using SAMtools, then processed with BCFtools [19] to identify variants, which are exported to VCF format. To evaluate correction accuracy while accounting for the inherent inconsistency in variant representation across different tools, we implemented the following protocol with 3 key steps: Step 1: Ground Truth Establishment: The simulated mutated genome is compared against the original input genome using compare_genomes.sh to generate a ground truth variant file (VCF file A); Step 2: Tool Output Analysis: The corrected genome produced by the correction tool is compared against the same original input genome using compare_genomes.sh to generate a prediction variant file (VCF file B); Step 3: Performance Assessment: The vcf_comparison.py utility compares a ground truth variant file (VCF file A) against a prediction variant file (VCF file B) using BCFtools isec to calculate key performance metrics. These include: true positives (TP), representing variants correctly identified; false positives (FP), representing variants incorrectly identified; false negatives (FN), representing missed variants; sensitivity, the proportion of actual variants correctly identified (TP/[TP + FN]); and precision, the proportion of identified variants that are correct (TP/[TP + FP]).

### Validation protocol with real NGS datasets

Paired-end Illumina reads from DRR119675, SRR1770413, and SRR10175772 were analyzed against BA000036.3, U00096.3, and NZ_LS483396.1, respectively. ProGenFixer was run with the default *k*-mer parameters, 3 threads, 3 iterations, and the --fix option. NextPolish v1.4.1 was run with paired short reads using max_depth = 120 and 3 threads. Each final FASTA was aligned to its matched original reference with minimap2 using the asm5 preset, and variants were normalized with paftools.js call. Exact shared variants required identical coordinates and REF/ALT alleles. Small-variant read support was calculated from the original-reference alignment with SAMtools after applying MAPQ >= 20 and base quality >= 20 filters. Depth and reference/alternate allele counts were evaluated for every tool-specific small variant. Sites with substantial support for both alleles were classified descriptively as mixed-support rather than assigned as correction errors; without an independent truth assembly, such evidence cannot distinguish strain heterogeneity, a composite or chimeric isolate, contamination, or mapping ambiguity. For the intended reference-update use case, retention of the curated allele at an ambiguous site was interpreted as a conservative decision. Wall-clock runtimes were measured in the same local analysis environment.

## Data Availability

ProGenFixer is freely available at https://github.com/Scilence2022/ProGenFixer.The real Illumina datasets analyzed in this study are available from the Sequence Read Archive under accessions DRR119675, SRR1770413, and SRR10175772; the corresponding reference accessions are BA000036.3, U00096.3, and NZ_LS483396.1.
