# Supplementary material for: ProGenFixer: An Ultrafast and Accurate Tool for Correcting Prokaryotic Genome Sequences Using a Mapping-Free Algorithm
Source: Comput Struct Biotechnol J. 2026 Aug 25;35(1):0183. doi: 10.34133/csbj.0183 (PMC13504041; doi:10.34133/csbj.0183)
Supplement: Supplementary file 1 [file csbj.0183.f1.docx]

# Supplementary materials

# ProGenFixer: an ultra-fast and accurate tool for correcting prokaryotic genome sequences using a mapping-free algorithm

**Authors:** Lifu Song^1,2,3,4*^, Mei Wang^2,3^, Xiaoping Liao^2,3,4,5^, Junli Wu^1,2^, Zhenkun Shi^2,3^, Ruoyu Wang^2,3^, Haoran Li^2,3^, Lulu Liu^2,3,4^, Botao He^2,3^, Xiaomeng Ni^2,3^, Qinggang Li^1^, Jinshan Li^6^, Hongwu Ma^2,3,4,5^, Ping Zheng^2,3,4,5^, Jibin Sun^2,3,4,5*^, Yanhe Ma^6,7^

*Correspondence to: Lifu Song: [songlf@tib.cas.cn](mailto:songlf@tib.cas.cn), Jibin Sun: [sun_jb@tib.cas.cn](mailto:sun_jb@tib.cas.cn)

# Supplementary Methods

## Default parameters of ProGenFixer

Unless otherwise stated, all analyses in this study used the default parameters of ProGenFixer. The k-mer length was set to k = 31; *k*-mers of this length are used to compare the input genome against the sequencing reads and to gate extension of candidate variation regions, so that a region is extended only while flanking anchor *k*-mers remain consistent between the reference and the reads.

Two coverage thresholds control the correction workflow. A candidate-region threshold, c = 3, flags low-support regions of the input genome as candidate variation sites; this comparatively permissive threshold favors sensitivity during detection. A separate anchor threshold, a = 8, governs anchor k-mer selection and acceptance of local variant paths during reconstruction of the corrected sequence; this higher threshold suppresses weak or spurious local paths and favors precision. Both thresholds are fixed defaults rather than coverage-adaptive values, and the current implementation does not include an automatic parameter-tuning mechanism. Under very low sequencing coverage these defaults may require manual adjustment, which we note as a limitation.

The corrected sequence content within each candidate variation region is estimated using a local alternative-assembly approach. To bound computational cost and avoid spurious reconstruction across long, low-complexity spans, the maximum local-assembly length was set to 1000 bp; candidate regions exceeding this length are reported rather than reconstructed. Correction was applied iteratively for three iterations, allowing newly resolved sequence to inform subsequent passes, and all runs used three threads. The --fix option was enabled so that confidently supported changes were written to the output genome, with the input reference otherwise retained under ProGenFixer's conservative reference-update strategy.

**Computational environment**

The simulated-data benchmarks were performed on a Linux workstation equipped with two Intel Xeon Platinum 8375C processors (2.90 GHz) and 1 TB of DDR4-3200 memory. The real-data analyses were performed on macOS 26.4.1 using an Apple M3 Max with 16 CPU cores and 128 GB of memory.

**Software versions and parameters**

This section documents the versions and command-line parameters of all software tools used to generate the simulated-data benchmarks. Every tool was run with three threads to ensure a comparable computational footprint, and, unless otherwise stated, all other parameters were left at their program defaults.

Simulated Illumina reads were generated with dwgsim v0.1.15 using a base error rate of 0.01 (-y 0.01), a read length of 150 bp for both mates (-1 150 -2 150), and haploid simulation (-H); the per-run mutation rate (-r), indel fraction (-R), indel extension probability (-X), and mean coverage (-C) were varied as described for each benchmark. These reads were then supplied to each correction tool in turn.

The remaining tools represent the comparison methods. For the BWA+GATK pipeline, reads were aligned with BWA-MEM v0.7.19-r1273 and processed with SAMtools v1.21 (sorting, fixmate, and duplicate marking), after which variants were called with GATK v4.6.1.0 (HaplotypeCaller) from the duplicate-marked, coordinate-sorted alignments. NextPolish v1.4.1 was run in short-read (sgs) mode with task = best, following the pipeline defined in run.cfg, with corrections derived from the same paired-end reads via BWA-MEM alignment. JASPER v1.0.4 was run with a *k*-mer length of 31 (-*k* 31) and three polishing passes (-p 3), and pypolca v0.3.1 was run with the --force option (-f) to overwrite existing output.

**Fig. S1 Runtime comparison of ProGenFixer with other genome correction tools.** The bar graph shows the run time (in seconds) taken to correct the *C. glutamicum* ATCC 13032 genome using various tools (ProGenFixer, BWA+GATK, Jasper, NextPolish, and Pypolca) at different read coverages of 100X and 400X. Runtimes are presented as the average of three independent repeats and the standard deviation values, which are too small to be visualized, are provided in **Table S3**.

**Fig. S2 Read-mapping support for the ProGenFixer-corrected deletion at U00096.3:257900-258675 in SRR1770413.**

Illumina reads from SRR1770413 were mapped to the genome sequence before and after ProGenFixer correction and visualized using CodeXomics (https://github.com/Scilence2022/CodeXomics). The original reference shows an unsupported interval spanning U00096.3:257900-258675, whereas the flanking regions are well covered. After ProGenFixer removes this interval, reads map continuously across the corrected junction, supporting the deletion call.

**Fig. S3 Read-mapping support for the ProGenFixer-corrected deletion at U00096.3:279163-290633 in SRR1770413.**

Illumina reads from SRR1770413 were mapped to the genome sequence before and after ProGenFixer correction and visualized using CodeXomics (https://github.com/Scilence2022/CodeXomics). The original reference contains a large low/no-coverage interval at U00096.3:279163-290633 with covered flanking regions. After ProGenFixer correction, reads support the new junction across the deleted segment, indicating that this reference interval is absent from the sequenced isolate.

**Fig. S4 Read-mapping support for the ProGenFixer-corrected insertion at NZ_LS483396.1:1680285 in SRR10175772.**

Illumina reads from SRR10175772 were mapped to the genome sequence before and after ProGenFixer correction and visualized using CodeXomics (https://github.com/Scilence2022/CodeXomics). In the original reference, reads show local discordance consistent with sequence missing at NZ_LS483396.1:1680285. After insertion of ACGTCCGGGTGCCGACCGTC by ProGenFixer, reads align coherently across the corrected locus, supporting the inserted sequence.

**Fig. S5 Read-mapping support for the ProGenFixer-corrected insertion at NZ_LS483396.1:1944707 in SRR10175772.**

Illumina reads from SRR10175772 were mapped to the genome sequence before and after ProGenFixer correction and visualized using CodeXomics (https://github.com/Scilence2022/CodeXomics). The original reference shows mapping inconsistency around NZ_LS483396.1:1944707, consistent with missing sequence. After ProGenFixer inserts TGCCCGCGGGATCTGTCCGCCCTGCTGGACCGC, read alignments become locally consistent across the corrected region, supporting the ProGenFixer correction.

**Table. S1** **Accuracy metrics of ProGenFixer compared to other genome correction tools for handling a mixture of substitutions and indels.**

Next-Generation Sequencing (NGS) reads were simulated to 100× coverage using *dwgsim* (v0.1.15) with the *C. glutamicum* ATCC 13032 genome sequence (GenBank accession BA000036.3) as the reference. Simulation parameters included an expected mutation rate of 0.3‰, an indel fraction of 0.1 (meaning 10% of mutations were indels), and an indel extension probability of 0.3.

|  | ProGenFixer | BWA+GATK | Jasper | NextPolish | Pypolca |
| --- | --- | --- | --- | --- | --- |
| True Positives | 950 | 714 | 921 | 960 | 956 |
| False Positives | 5 | 1 | 1 | 1 | 0 |
| False Negatives | 22 | 258 | 51 | 12 | 16 |
| Sensitivity | 97.74% | 73.46% | 94.75% | 98.77% | 98.35% |
| Precision | 99.48% | 99.86% | 99.89% | 99.90% | 100.00% |

*Note: TP = True Positives, FP = False Positives, FN = False Negatives*

**Table. S2** **Speed Evaluation details of ProGenFixer in correcting the *E. coli* MG1655 genome sequence in comparison with other tools.**

NGS reads were simulated using *dwgsim* (version 0.1.15) with the *E. coli* MG1655 genome sequence (LR881938.1).

| NGS reads coverage = 100 | | | | | | | | | | | |
| --- | --- | --- | --- | --- | --- | --- | --- | --- | --- | --- | --- |
| Tools | **Average Run time (s)** | **SD.** | **Real** | **User** | **Sys** | **Real** | **User** | **Sys** | **Real** | **User** | **Sys** |
| BWA+GATK | 367.40 | 8.16 | 361.95 | 884.62 | 44.02 | 376.79 | 917.35 | 39.66 | 363.47 | 868.56 | 34.31 |
| ProGenFixer | 12.38 | 0.38 | 12.79 | 35.33 | 2.15 | 12.31 | 34.37 | 1.99 | 12.04 | 34.03 | 2.01 |
| Jasper | 71.93 | 0.31 | 72.24 | 213.48 | 9.78 | 71.63 | 210.60 | 9.39 | 71.93 | 210.78 | 9.81 |
| NextPolish | 482.51 | 1.13 | 481.27 | 1362.82 | 26.88 | 483.47 | 1370.09 | 27.47 | 482.78 | 1369.21 | 27.88 |
| Pypolca | 209.58 | 2.01 | 208.63 | 423.59 | 6.06 | 208.22 | 421.99 | 5.70 | 211.89 | 428.50 | 6.02 |
| NGS reads coverage = 400 | | | | | | | | | | | |
| Tools | **Average Run time (s)** | **SD.** | **Real** | **User** | **Sys** | **Real** | **User** | **Sys** | **Real** | **User** | **Sys** |
| ProGenFixer | 49.13 | 7.67 | 44.88 | 138.84 | 6.63 | 57.98 | 154.18 | 16.98 | 44.52 | 139.80 | 6.84 |
| BWA+GATK | 1206.47 | 14.63 | 1194.66 | 2596.72 | 115.72 | 1222.83 | 2629.48 | 99.30 | 1201.91 | 2564.18 | 81.99 |
| Jasper | 251.37 | 4.39 | 252.94 | 760.75 | 30.97 | 254.76 | 761.13 | 31.62 | 246.41 | 745.31 | 30.99 |
| NextPolish | 1729.12 | 2.64 | 1729.48 | 5427.61 | 109.28 | 1726.31 | 5422.27 | 105.78 | 1731.56 | 5439.19 | 106.84 |
| Pypolca | 846.33 | 0.90 | 845.34 | 1719.43 | 30.00 | 847.09 | 1719.11 | 25.93 | 846.55 | 1710.17 | 26.59 |

**Table. S3** **Evaluation details of ProGenFixer in correcting the *C. glutamicum* ATCC 13032 genome sequence in comparison with other tools.**

NGS reads were simulated using *dwgsim* (v0.1.15) with the *C. glutamicum* ATCC 13032 genome sequence (GenBank accession BA000036.3) as the reference.

| NGS reads coverage = 100 | | | | | | | | | | | |
| --- | --- | --- | --- | --- | --- | --- | --- | --- | --- | --- | --- |
| Tools | **Average Run time (s)** | **SD.** | **Real** | **User** | **Sys** | **Real** | **User** | **Sys** | **Real** | **User** | **Sys** |
| ProGenFixer | 10.16 | 0.26 | 10.20 | 29.08 | 2.21 | 10.40 | 29.36 | 2.21 | 9.88 | 27.42 | 2.08 |
| BWA+GATK | 274.55 | 4.07 | 278.93 | 718.57 | 33.98 | 270.89 | 691.30 | 38.80 | 273.83 | 698.59 | 35.85 |
| Jasper | 49.74 | 6.42 | 45.72 | 135.97 | 6.56 | 46.35 | 136.98 | 6.17 | 57.15 | 167.25 | 7.00 |
| NextPolish | 362.29 | 0.37 | 362.25 | 978.16 | 20.42 | 361.95 | 977.08 | 20.79 | 362.68 | 977.07 | 20.14 |
| Pypolca | 148.80 | 0.56 | 149.41 | 302.71 | 4.23 | 148.70 | 299.54 | 4.04 | 148.30 | 298.16 | 4.07 |
| NGS reads coverage = 400 | | | | | | | | | | | |
| Tools | **Average Run time (s)** | **SD.** | **Real** | **User** | **Sys** | **Real** | **User** | **Sys** | **Real** | **User** | **Sys** |
| ProGenFixer | 31.46 | 1.84 | 38.78 | 110.86 | 9.92 | 33.58 | 108.72 | 5.46 | 30.40 | 94.72 | 4.82 |
| BWA+GATK | 874.59 | 3.36 | 858.79 | 1893.85 | 91.51 | 870.71 | 1910.61 | 83.88 | 876.53 | 1953.60 | 84.35 |
| Jasper | 229.12 | 114.60 | 158.40 | 483.74 | 20.52 | 361.45 | 974.80 | 20.81 | 162.95 | 492.64 | 23.58 |
| NextPolish | 1252.82 | 0.01 | 1252.79 | 3884.96 | 76.89 | 1252.82 | 3883.79 | 86.48 | 1252.84 | 3884.18 | 83.37 |
| Pypolca | 597.06 | 0.50 | 598.72 | 1210.38 | 17.42 | 596.49 | 1204.30 | 19.12 | 597.35 | 1208.63 | 16.93 |

**Table. S4 Performance of ProGenFixer in handling indels of varying lengths using *C. glutamicum* ATCC 13032 genome as the reference.**

NGS reads (100× coverage) were simulated using *dwgsim* (v0.1.15) with the *C. glutamicum* ATCC 13032 genome (GenBank: BA000036.3) as the reference. To evaluate performance specifically on indels, only indel errors were introduced by setting the indel fraction parameter *-R* to 1. The expected indel lengths were varied by adjusting the indel extension probability using the *-X* option in *dwgsim*. The method for calculating expected indel lengths from different *-X* values is detailed in the **Materials and Methods** section.

|  | ProGenFixer | BWA+GATK | Jasper | NextPolish | Pypolca | Indel length |
| --- | --- | --- | --- | --- | --- | --- |
| TP | 893 | 186 | 805 | 904 | 898 | ~1.11 bp  *-X 0.1* |
| FP | 0 | 1 | 0 | 0 | 0 |  |
| FN | 24 | 731 | 112 | 13 | 19 |  |
| Sensitivity | 97.38% | 20.28% | 87.79% | 98.58% | 97.93% |  |
| Precision | 100.00% | 99.47% | 100.00% | 100.00% | 100.00% |  |
| TP | 889 | 187 | 616 | 905 | 896 | ~1.43 bp  *-X 0.3* |
| FP | 1 | 1 | 1 | 0 | 0 |  |
| FN | 23 | 725 | 296 | 7 | 16 |  |
| Sensitivity | 97.48% | 20.50% | 67.54% | 99.23% | 98.25% |  |
| Precision | 99.89% | 99.47% | 99.84% | 100.00% | 100.00% |  |
| TP | 898 | 191 | 448 | 913 | 905 | ~2 bp  *-X 0.5* |
| FP | 0 | 2 | 2 | 1 | 0 |  |
| FN | 25 | 732 | 475 | 10 | 18 |  |
| Sensitivity | 97.29% | 20.69% | 48.54% | **98.92%** | **98.05%** |  |
| Precision | 100.00% | 98.96% | 99.56% | **99.89%** | **100.00%** |  |
| TP | 906 | 199 | 290 | 926 | 913 | ~3.33 bp  *-X 0.7* |
| FP | 1 | 0 | 2 | 0 | 0 |  |
| FN | 33 | 740 | 649 | 13 | 26 |  |
| Sensitivity | **96.49%** | 21.19% | 30.88% | 98.62% | 97.23% |  |
| Precision | **99.89%** | 100.00% | 99.32% | 100.00% | 100.00% |  |
| TP | 958 | 206 | 109 | 945 | 915 | ~10 bp  *-X 0.9* |
| FP | 0 | 7 | 1 | 16 | 21 |  |
| FN | 26 | 778 | 875 | 39 | 69 |  |
| Sensitivity | **97.36%** | 20.93% | 11.08% | 96.04% | 92.99% |  |
| Precision | **100.00%** | 96.71% | 99.09% | 98.34% | 97.76% |  |
| TP | 884 | 216 | 50 | 859 | 790 | ~20 bp  *-X 0.95* |
| FP | 12 | 5 | 3 | 7 | 13 |  |
| FN | 22 | 690 | 856 | 47 | 116 |  |
| Sensitivity | **97.57%** | 23.84% | 5.52% | 94.81% | 87.20% |  |
| Precision | **98.66%** | 97.74% | 94.34% | 99.19% | 98.38% |  |

*Note: TP = True Positives, FP = False Positives, FN = False Negatives*

**Table. S5** **Performance of ProGenFixer in handling substitutions.**

NGS reads were simulated to 100× coverage using *dwgsim* (v0.1.15) with the *C. glutamicum* ATCC 13032 genome sequence (GenBank accession BA000036.3) as the reference. To evaluate performance specifically on substitutions, only substitution errors were introduced (indel fraction set to 0), with various mutation rates specified using the *-r* option in dwgsim.

|  | ProGenFixer | BWA+GATK | Jasper | NextPolish | Pypolca | Mutation  rate |
| --- | --- | --- | --- | --- | --- | --- |
| TP | 353 | 289 | 357 | 362 | 361 | 0.1‰ |
| FP | 4 | 0 | 0 | 1 | 0 |  |
| FN | 10 | 74 | 6 | 1 | 2 |  |
| Sensitivity | 97.25% | 79.61% | 98.35% | 99.72% | 99.45% |  |
| Precision | 98.88% | 100.00% | 100.00% | 99.72% | 100.00% |  |
| TP | 911 | 746 | 919 | 928 | 922 | 0.3‰ |
| FP | 0 | 2 | 0 | 0 | 0 |  |
| FN | 30 | 195 | 22 | 13 | 19 |  |
| Sensitivity | 96.81% | 79.28% | 97.66% | 98.62% | 97.98% |  |
| Precision | 100.00% | 99.73% | 100.00% | 100.00% | 100.00% |  |
| TP | 1603 | 1308 | 1605 | 1634 | 1618 | 0.5‰ |
| FP | 3 | 1 | 1 | 1 | 0 |  |
| FN | 45 | 340 | 43 | 14 | 30 |  |
| Sensitivity | 97.27% | 79.37% | 97.39% | 99.15% | 98.18% |  |
| Precision | 99.81% | 99.92% | 99.94% | 99.94% | 100.00% |  |
| TP | 2310 | 1851 | 2324 | 2363 | 2342 | 0.7‰ |
| FP | 11 | 6 | 1 | 1 | 0 |  |
| FN | 74 | 533 | 60 | 21 | 42 |  |
| Sensitivity | 96.90% | 77.64% | 97.48% | 99.12% | 98.24% |  |
| Precision | 99.53% | 99.68% | 99.96% | 99.96% | 100.00% |  |
| TP | 2928 | 2397 | 2929 | 2988 | 2961 | 0.9‰ |
| FP | 8 | 3 | 2 | 0 | 0 |  |
| FN | 85 | 616 | 84 | 25 | 52 |  |
| Sensitivity | 97.18% | 79.56% | 97.21% | 99.17% | 98.27% |  |
| Precision | 99.73% | 99.88% | 99.93% | 100.00% | 100.00% |  |
| TP | 3231 | 2658 | 3252 | 3316 | 3279 | 1‰ |
| FP | 7 | 3 | 1 | 0 | 0 |  |
| FN | 111 | 684 | 90 | 26 | 63 |  |
| Sensitivity | 96.68% | 79.53% | 97.31% | 99.22% | 98.11% |  |
| Precision | 99.78% | 99.89% | 99.97% | 100.00% | 100.00% |  |

*Note: TP = True Positives, FP = False Positives, FN = False Negatives*

**Table. S6** **Tool-specific normalized variants and read support in real Illumina resequencing datasets.**

ProGenFixer and NextPolish were run on the same paired-end reads for *E. coli* K-12 MG1655 (SRR1770413; reference U00096.3) and *Micrococcus luteus* NCTC 2665 (SRR10175772; reference NZ_LS483396.1). Final corrected sequences were independently aligned to the original reference, and normalized variants specific to either tool are listed. Depth, reference allele count, alternate allele count, and alternate allele fraction were calculated from the original-reference read alignment where applicable. For large deletion intervals, the coordinate span is shown and depth represents the mean coverage across the interval; allele counts are not applicable. Shared variants are not shown in this table.

| Dataset | Tool | Chrom | Position | Type | Ref allele/display | Alt allele/display | Ref length | Alt length | Depth | Ref count | Alt count | Alt fraction |
| --- | --- | --- | --- | --- | --- | --- | --- | --- | --- | --- | --- | --- |
| SRR1770413 | ProGenFixer | U00096.3 | 257900 | DEL interval | 257900-258675 | deleted | 13 | 7 | 0.40 | / | / | / |
| SRR1770413 | ProGenFixer | U00096.3 | 279163 | DEL interval | 279163-290633 | deleted | 13 | 7 | 0.99 | / | / | / |
| SRR1770413 | NextPolish | U00096.3 | 257911 | SNP | A | T | 1 | 1 | 3.00 | 2 | 1 | 33.3%% |
| SRR1770413 | NextPolish | U00096.3 | 257923 | SNP | T | G | 1 | 1 | 6.00 | 5 | 1 | 16.70% |
| SRR1770413 | NextPolish | U00096.3 | 578729 | SNP | A | G | 1 | 1 | 129.00 | 63 | 66 | 51.16% |
| SRR1770413 | NextPolish | U00096.3 | 578831 | SNP | T | C | 1 | 1 | 126.00 | 61 | 65 | 51.59% |
| SRR1770413 | NextPolish | U00096.3 | 579127 | SNP | T | C | 1 | 1 | 116.00 | 52 | 64 | 55.17% |
| SRR1770413 | NextPolish | U00096.3 | 579134 | SNP | T | C | 1 | 1 | 116.00 | 51 | 65 | 56.03% |
| SRR1770413 | NextPolish | U00096.3 | 579146 | SNP | A | G | 1 | 1 | 114.00 | 51 | 63 | 55.26% |
| SRR1770413 | NextPolish | U00096.3 | 579271 | SNP | G | A | 1 | 1 | 118.00 | 59 | 59 | 50.00% |
| SRR1770413 | NextPolish | U00096.3 | 579285 | SNP | T | C | 1 | 1 | 115.00 | 58 | 57 | 49.57% |
| SRR1770413 | NextPolish | U00096.3 | 579502 | SNP | A | G | 1 | 1 | 113.00 | 51 | 62 | 54.87% |
| SRR1770413 | NextPolish | U00096.3 | 579540 | SNP | T | C | 1 | 1 | 119.00 | 56 | 63 | 52.94% |
| SRR1770413 | NextPolish | U00096.3 | 579651 | SNP | C | T | 1 | 1 | 130.00 | 64 | 66 | 50.77% |
| SRR1770413 | NextPolish | U00096.3 | 579717 | SNP | A | C | 1 | 1 | 136.00 | 70 | 66 | 48.53% |
| SRR1770413 | NextPolish | U00096.3 | 579811 | SNP | C | T | 1 | 1 | 128.00 | 62 | 66 | 51.56% |
| SRR1770413 | NextPolish | U00096.3 | 580020 | SNP | G | A | 1 | 1 | 112.00 | 59 | 53 | 47.32% |
| SRR1770413 | NextPolish | U00096.3 | 580302 | SNP | C | T | 1 | 1 | 83.00 | 56 | 27 | 32.53% |
| SRR1770413 | NextPolish | U00096.3 | 696470 | SNP | C | T | 1 | 1 | 58.00 | 55 | 41 | 100.00% |
| SRR1770413 | NextPolish | U00096.3 | 1634328 | SNP | T | C | 1 | 1 | 49.00 | 40 | 9 | 18.37% |
| SRR1770413 | NextPolish | U00096.3 | 1634337 | SNP | C | A | 1 | 1 | 51.00 | 42 | 9 | 17.65% |
| SRR1770413 | NextPolish | U00096.3 | 1634338 | SNP | T | G | 1 | 1 | 51.00 | 42 | 9 | 17.65% |
| SRR1770413 | NextPolish | U00096.3 | 1634349 | SNP | C | T | 1 | 1 | 52.00 | 43 | 9 | 17.31% |
| SRR1770413 | NextPolish | U00096.3 | 1634370 | SNP | T | C | 1 | 1 | 49.00 | 39 | 10 | 20.41% |
| SRR1770413 | NextPolish | U00096.3 | 1634382 | SNP | T | C | 1 | 1 | 53.00 | 43 | 10 | 18.87% |
| SRR1770413 | NextPolish | U00096.3 | 1634388 | SNP | T | G | 1 | 1 | 51.00 | 41 | 10 | 19.61% |
| SRR1770413 | NextPolish | U00096.3 | 1634404 | SNP | A | G | 1 | 1 | 53.00 | 42 | 11 | 20.75% |
| SRR1770413 | NextPolish | U00096.3 | 1634482 | SNP | T | C | 1 | 1 | 58.00 | 43 | 15 | 25.86% |
| SRR1770413 | NextPolish | U00096.3 | 1634484 | SNP | G | T | 1 | 1 | 57.00 | 42 | 15 | 26.32% |
| SRR1770413 | NextPolish | U00096.3 | 1634569 | SNP | A | T | 1 | 1 | 64.00 | 40 | 24 | 37.50% |
| SRR1770413 | NextPolish | U00096.3 | 1634571 | SNP | C | T | 1 | 1 | 66.00 | 42 | 24 | 36.36% |
| SRR1770413 | NextPolish | U00096.3 | 1634578 | SNP | T | C | 1 | 1 | 68.00 | 41 | 27 | 39.71% |
| SRR1770413 | NextPolish | U00096.3 | 1634597 | SNP | C | A | 1 | 1 | 66.00 | 40 | 26 | 39.39% |
| SRR1770413 | NextPolish | U00096.3 | 1634658 | SNP | A | G | 1 | 1 | 66.00 | 38 | 28 | 42.42% |
| SRR1770413 | NextPolish | U00096.3 | 1634661 | SNP | C | G | 1 | 1 | 67.00 | 40 | 27 | 40.30% |
| SRR1770413 | NextPolish | U00096.3 | 1634676 | SNP | A | G | 1 | 1 | 67.00 | 39 | 28 | 41.79% |
| SRR1770413 | NextPolish | U00096.3 | 1634699 | SNP | A | C | 1 | 1 | 68.00 | 40 | 28 | 41.18% |
| SRR1770413 | NextPolish | U00096.3 | 1634724 | SNP | G | A | 1 | 1 | 62.00 | 37 | 25 | 40.32% |
| SRR1770413 | NextPolish | U00096.3 | 1634769 | SNP | T | A | 1 | 1 | 64.00 | 40 | 24 | 37.50% |
| SRR1770413 | NextPolish | U00096.3 | 1634778 | SNP | T | G | 1 | 1 | 62.00 | 39 | 23 | 37.10% |
| SRR1770413 | NextPolish | U00096.3 | 1634799 | SNP | A | G | 1 | 1 | 61.00 | 38 | 23 | 37.70% |
| SRR1770413 | NextPolish | U00096.3 | 1636484 | SNP | C | T | 1 | 1 | 75.00 | 46 | 29 | 38.67% |
| SRR1770413 | NextPolish | U00096.3 | 1636503 | SNP | T | C | 1 | 1 | 76.00 | 45 | 31 | 40.79% |
| SRR1770413 | NextPolish | U00096.3 | 1690156 | SNP | C | A | 1 | 1 | 52.00 | 2 | 50 | 96.15% |
| SRR1770413 | NextPolish | U00096.3 | 2003266 | SNP | T | A | 1 | 1 | 56.00 | 28 | 28 | 50.00% |
| SRR1770413 | NextPolish | U00096.3 | 2768847 | SNP | C | T | 1 | 1 | 442.00 | 46 | 396 | 89.59% |
| SRR1770413 | NextPolish | U00096.3 | 2769114 | SNP | T | A | 1 | 1 | 455.00 | 44 | 410 | 90.11% |
| SRR1770413 | NextPolish | U00096.3 | 2769120 | SNP | G | C | 1 | 1 | 455.00 | 45 | 409 | 89.89% |
| SRR1770413 | NextPolish | U00096.3 | 3763136 | SNP | A | C | 1 | 1 | 34.00 | 15 | 19 | 55.89% |
| SRR1770413 | NextPolish | U00096.3 | 4013117 | SNP | C | T | 1 | 1 | 55.00 | 16 | 39 | 70.91% |
| SRR1770413 | NextPolish | U00096.3 | 4166470 | SNP | C | T | 1 | 1 | 59.00 | 4 | 55 | 93.22% |
| SRR1770413 | NextPolish | U00096.3 | 4248820 | SNP | T | A | 1 | 1 | 48.00 | 2 | 46 | 95.83% |
| SRR10175772 | ProGenFixer | NZ_LS483396.1 | 1680285 | INS | A | ACGTCCGGGTGCCGACCGTC | 1 | 20 | / | / | / | / |
| SRR10175772 | ProGenFixer | NZ_LS483396.1 | 1944707 | INS | T | TGCCCGCGGGATCTGTCCGCCCTGCTGGACCGC | 1 | 33 | / | / | / | / |
| SRR10175772 | NextPolish | NZ_LS483396.1 | 120103 | SNP | T | A | 1 | 1 | 4.00 | 2 | 2 | 50.00% |
| SRR10175772 | NextPolish | NZ_LS483396.1 | 579851 | SNP | C | G | 1 | 1 | 2.00 | 1 | 1 | 50.00% |
| SRR10175772 | NextPolish | NZ_LS483396.1 | 584585 | SNP | C | G | 1 | 1 | 1.00 | 0 | 1 | 100.00% |
| SRR10175772 | NextPolish | NZ_LS483396.1 | 1065454 | SNP | A | G | 1 | 1 | 2.00 | 1 | 1 | 50.00% |
| SRR10175772 | NextPolish | NZ_LS483396.1 | 1072498 | SNP | G | T | 1 | 1 | 3.00 | 2 | 1 | 33.30% |
| SRR10175772 | NextPolish | NZ_LS483396.1 | 1075753 | SNP | C | G | 1 | 1 | 1.00 | 0 | 1 | 100.00% |
| SRR10175772 | NextPolish | NZ_LS483396.1 | 1078107 | SNP | A | G | 1 | 1 | 1.00 | 0 | 1 | 100.00% |
| SRR10175772 | NextPolish | NZ_LS483396.1 | 1078112 | SNP | A | G | 1 | 1 | 1.00 | 0 | 1 | 100.00% |
| SRR10175772 | NextPolish | NZ_LS483396.1 | 1078134 | SNP | T | G | 1 | 1 | 1.00 | 0 | 1 | 100.00% |
| SRR10175772 | NextPolish | NZ_LS483396.1 | 1078156 | SNP | C | G | 1 | 1 | 0.00 | 0 | 1 | 100.00% |
| SRR10175772 | NextPolish | NZ_LS483396.1 | 1079629 | SNP | G | A | 1 | 1 | 7.00 | 0 | 7 | 100.00% |
| SRR10175772 | NextPolish | NZ_LS483396.1 | 1089953 | SNP | G | A | 1 | 1 | 1.00 | 0 | 1 | 100.00% |
| SRR10175772 | NextPolish | NZ_LS483396.1 | 1099913 | INS | G | GCGTC | 1 | 5 | 3.00 | 0 | 3 | 100.00% |
| SRR10175772 | NextPolish | NZ_LS483396.1 | 1104806 | SNP | G | T | 1 | 1 | 0.00 | 0 | 1 | 100.00% |
| SRR10175772 | NextPolish | NZ_LS483396.1 | 1246601 | SNP | C | G | 1 | 1 | 0.00 | 0 | 1 | 100.00% |
| SRR10175772 | NextPolish | NZ_LS483396.1 | 1246602 | SNP | C | G | 1 | 1 | 0.00 | 0 | 1 | 100.00% |
| SRR10175772 | NextPolish | NZ_LS483396.1 | 1485256 | SNP | C | T | 1 | 1 | 13.00 | 0 | 13 | 100.00% |
| SRR10175772 | NextPolish | NZ_LS483396.1 | 2233494 | SNP | G | A | 1 | 1 | 63.00 | 27 | 36 | 57.14% |
| SRR10175772 | NextPolish | NZ_LS483396.1 | 2459119 | SNP | T | G | 1 | 1 | 3.00 | 2 | 1 | 33.33% |

**Table. S7 Repeat- versus unique-context classification of corrected calls in real Illumina resequencing datasets.**

Repeat/unique context was assigned using local 31-mer uniqueness in the corresponding reference genome; the majority of corrected calls fall in unique regions.

| Dataset | Reference | Tool | Repeat-associated calls | Total calls |
| --- | --- | --- | --- | --- |
| DRR119675 | *C. glutamicum* ATCC 13032 | ProGenFixer | 2 | 79 |
| DRR119675 | *C. glutamicum* ATCC 13032 | NextPolish | 2 | 79 |
| SRR1770413 | *E. coli* K-12 MG1655 | ProGenFixer | 4 | 47 |
| SRR1770413 | *E. coli* K-12 MG1655 | NextPolish | 29 | 95 |
| SRR10175772 | *M. luteus* NCTC 2665 | ProGenFixer | 2 | 40 |
| SRR10175772 | *M. luteus* NCTC 2665 | NextPolish | 2 | 57 |
